# “If you don’t actually care for somebody, how can you help them?”: Exploring Young People’s Core Needs in Mental Healthcare—Directions for Improving Service Provision

**DOI:** 10.1007/s10597-024-01237-y

**Published:** 2024-03-02

**Authors:** Louise Lynch, Anne Moorhead, Maggie Long, Isobel Hawthorne-Steele

**Affiliations:** 1 School of Communication and Media, Faculty of Arts, Humanities and Social Sciences, Ulster University, York Street, Belfast, Co. Antrim, BT15 1ED Northern Ireland; 2 School of Communication and Media, Institute for Nursing and Health Research, Ulster University, York Street, Belfast, Co. Antrim, BT15 1ED Northern Ireland; 3 School of Communication and Media, Centre for Communication and Media Research, Faculty of Arts, Humanities and Social Science, Ulster University, York Street, Belfast, Co. Antrim, BT15 1ED Northern Ireland; 4 School of Applied Social and Policy Sciences, Faculty of Arts, Humanities and Social Sciences, Ulster University, York Street, Belfast, Co. Antrim, BT15 1ED Northern Ireland

**Keywords:** Young people, Mental health services, Help-seeking, Healthcare needs, Lived experience

## Abstract

Youth suicide and mental health are important issues of global concern that require timely and evidence-based interventions to increase quality of life and prevent deaths. Findings report that young people have lower mental health help-seeking rates, and there is a lack of qualitative research examining why. The aim of this research study was to further understanding on young people’s *core needs* in mental healthcare based on actual experiences (PLE) of help-seeking with providers of mental health services. Constructivist Grounded Theory methods (Charmaz, 2014) informed this study design, and in-depth interviews and a focus group were conducted with 18 young people. The findings were presented across *four* sub-categories, which together describe the common factors, that are regarded as essential in youth mental healthcare provision. These include: 1. *The services;* 2. *The helper;* 3. *The interventions,* and 4. *The impact of development.* Critical discussion into young people’s needs in mental healthcare was provided including the key *service* factors*, approach and rapport* with helpers*,* types of *intervention* and alignment with typical *developmental* capacity. This article provides guidance on how to improve, design, or reform service provision, and can be a useful resource for policy makers, service providers and practitioners. This study concluded that youth participation in the co-design of service provision is important as it can reduce health disparities and ensure that services provide relevant, respectful and suitable care that reflects the way in which young people experience mental health problems as well as the ways in which they want to be helped.

## Introduction

Mental health conditions are generally recognized as first emerging early in the lifespan, between the ages of 4–35 years (Solmi et al., 2022; Kessler et al., 2007), and it is estimated that between 10 and 20% of young people have mental health problems but that the true prevalence rates are unknown (World Health Organisation (WHO), 2021). Mental health conditions in youth contribute to lower quality of life, disability, educational difficulties (Bilsen, 2018; Patel et al., 2007; Pompili, 2018; WHO, 2021) and suicide, which is the second leading cause of death for young people aged 15–29 years (WHO, 2023) and considered preventable with timely and evidence-based interventions. As such, early intervention in mental health is a healthcare topic of global priority (WHO, 2023) and increasing understanding in *help-seeking behaviour,* specifically, how to engage and facilitate young people to access interventions is a critical topic (Goodwin et al., 2016; Gulliver et al., 2010).

### Young People, Mental Health and Help-Seeking

Help-seeking can be described as a coping-mechanism (Chan, 2013), where an individual intentionally acts to solve a problem (Cornally & McCarthy, 2011) and involves the helper, the task and the recipient (Nadler, 1987). There exists a large body of international research that reports findings on the factors that can influence mental health help-seeking behaviours in young people, and these are predominantly described in the form of barriers or facilitators (Radez et al., 2021). Articles have consistently highlighted important *practical barriers,* detailing service access and suitability concerns, financial barriers, the absence of provision in rural areas (Hernan et al., 2010; Radez et al., 2021) or inaccessible and unresponsive services (Westberg et al., 2022). The *social factors* discussed as directly affecting youth mental health help-seeking include stigma, community attitudes and cultural expressions of distress (Byrow et al., 2020; Goodwin et al., 2016; Gulliver et al., 2010; Lynch et al., 2018; Michelmore & Hindley, 2012; Nam et al., 2010; Rowe et al., 2014). Friends and family are often reported as the preferred sources of help for mental health problems (Michelmore & Hindley, 2012; Rickwood et al., 2005; Rowe et al., 2014) and can act as significant barriers or facilitators in professional help-seeking (Lynch et al., 2023). Gatekeepers such as teachers, youth workers, or GPs are also important in supporting access to professional help (Leavey et al., 2011; Quinn et al., 2009; Rickwood et al., 2005, 2007), especially for young people with migrant or refugee experiences (De Anstiss et al., 2009; Ellis et al., 2010) or those experiencing homelessness (Collins & Barker, 2009; Crosby et al., 2018). With regard to *personal factors*, common findings include the role of self-management or the prioritising of self-reliance (Bramesfeld et al., 2006; Burlaka et al., 2014; Loureiro et al., 2013; Westberg et al., 2022); mental health literacy (Pearson & Hyde, 2021); or beliefs and attitudes towards mental healthcare (Chen et al., 2014; Dogra et al., 2012; Eisenberg et al., 2011; Jorm et al., 2008; Klineberg et al., 2011; Rickwood et al., 2005; Rickwood et al., 2007; Rothi & Leavey, 2006; Pheko et al., 2013; Pumpa & Martin, 2015, Wang et al., 2020). Indeed, the role of young people’s beliefs and attitudes towards mental health professionals and services is a greatly researched topic across global mental health literature and some authors suggest that while negative attitudes have a role in professional help negation, they do not solely prevent nor predict help-seeking (Eisenberg et al., 2012; Pearson & Hyde, 2021). Furthermore, many findings suggest that positive attitudes and future positive intentions to seek-help can be linked with previous positive experiences of help-seeking (Andriessen et al. 2018; Rickwood et al., 2005; Ryan et al., 2014; Rowe et al., 2014; Wilson & Deane, 2012). When problem-solving for a mental health concern, drawing on past experiences, approaches and expectations can inform help-seeking decisions (Chan, 2013; Rothi & Leavey, 2006) and research supports that previous personal and vicarious help-seeking experiences have been found to be highly impactful on young people (Charman et al., 2010; Gilchrist & Sullivan, 2006; Rowe et al., 2014; Ryan et al., 2014; Wilson & Deane, 2012). In particular, young people have reported experiences of poor-quality professional support including confidentiality breaches and developmentally inappropriate service provision (Charman et al., 2010; Damian et al., 2018; Gilchrist & Sullivan, 2006; Jones et al., 2017; Persson et al., 2017) as well as inadequate support for young people from migrant or refugee backgrounds (DeAnstiss & Ziaian, 2010; Byrow et al., 2020) or for those who identify as LGBTI + (Fish, 2020), all of which can discourage them and others they know from seeking help in the future.

In summation, the research on youth mental health help-seeking is substantial and predominantly quantitative, which has mapped the breadth of the topic categorically and provided important insight and interesting conclusions, but which also has limitations. Many research studies have used hypothetical scenarios or survey instruments and there is evidence to suggest that investigating intentions to seek help cannot predict or be translated into actual help-seeking behaviour (Eisenberg et al., 2012; Hughes & Huby, 2004). Therefore, qualitative research is essential with young people with lived experiences (PLE) regarding their mental health and associated help-seeking pathways (Law et al. 2020; Lynch et al., 2023). In addition, the negative labelling of young people as reluctant, resistant or unwilling can obscure the complex cost–benefit assessment processes (Chan, 2013) that result in non-help-seeking or early treatment exits. This can distract attention from the role of services in providing appropriate healthcare (Jones et al., 2017; Persson et al., 2017) and result in missed opportunities for service improvement. Thus, there is a distinct need for new directions and in-depth inquiry with mental health service users (Law et al. 2020; Lynch et al., 2023), as increasing understanding of experiences can inform measures to reduce health disparities affecting young people and support practitioners and services to better engage, facilitate and keep young people in mental healthcare (Breslin et al., 2022; Medlow et al., 2010; Raviv et al., 2009; Rughani et al., 2011).

### Aim and Scope of This Study

The research question asked *how can mental health service providers meet young people’s needs for mental healthcare?* Accordingly, the aim of this research study was to improve understanding on young people’s *core needs* in mental healthcare through evaluative inquiry of their help-seeking experiences to providers of mental healthcare. This research sought to consult directly with young people about their lived experiences of being young and asking for help with a mental health problem, to identify their healthcare needs and to support the improvement of mental health services, which can contribute towards quality of life and suicide prevention (O’Neill et al., 2018; WHO, 2023).

### Key Terms

Youth is a continuously evolving concept and definitions can be dependent on the economic conditions of a particular region (Arnett, 2023; UNESCO, 2010). In this research, the term, “young people” or “youth” are used interchangeably to refer to individuals in the age range of 10 to 25 years approximately, and adolescence and emerging adulthood are distinguished between where possible. The term “mental health problem” is used throughout to refer to the spectrum of personal distress and mental conditions that can affect an individual (Lynch et al., 2021). The term “help-seeking” is used to describe the actions of a young person when seeking external support with the aim of lowering their mental health distress. Finally, the term “LGBTI + ” refers to individuals who identify as Lesbian, Gay, Bisexual, Transgender or Intersex, which is the most commonly used acronym throughout Ireland (Department of Children, Equality, Disability, Integration & Youth, 2023).

## Methods

### Research Design

This qualitative study required a systematic and focused yet flexible toolset for conducting research with young people about their experiences and perspectives on help-seeking for a mental health problem. As the nature of this topic involves a complex social phenomena (Chan, 2013), Kathy Charmaz’s *Constructivist Grounded Theory* methods (GCT) (2014) were chosen for their ability to support the collection and management of rich data obtained through interviews and focus groups. These methods facilitated the exploration of the personal, social, structural, historical, and cultural meanings that shape individual experiences of mental health and help-seeking (Charmaz, 2014; Golafshani, 2003; MacKenzie & Knipe, 2006) and also in identifying the common experiences that present as needs from the shared experience of being young and help-seeking for psychological distress.

### Participants

The participants’ data in this research were part of a larger study on help-seeking that included mental health practitioners’ perspectives (n = 6). As this research explored healthcare needs for young people with lived experience of mental health help-seeking, only data provided by young people were included. The participants were young people aged 16–25 years from the Northwest of Ireland who had made at least one attempt to seek help for a mental health problem from a service provider. Help-seeking episodes that were attempted, partially completed, fully completed, or exited early were included. Participants self-reported how they met the selection criteria which are detailed in full in Table 1.

**Table 1:** Participant selection criteria

Inclusion	Exclusion
Aged between 16 and 25 years	Have not sought help for a mental health problem with any service
Have sought help with a formal service or semi-formal service with a minimum of one contact	Have been referred directly by a practitioner/service employee to the study
Have sought help with a formal or semi-formal service within the previous four years	Have an intellectual disability
Are not currently in crisis or in the early stages of receiving mental health support	Not able to provide consent

*Formal mental health services* were service providers and professionals who have a specified role in the delivery of mental health care such as counsellors, psychologists, psychiatrists and mental health nurses. *Semi-formal mental health services* were providers and professionals that do not have a specified role in delivery of mental health care but who encounter or provide support with those who need mental health care, typically school guidance counsellors and youth workers. A “helper” in the findings and discussion sections thus refers to an individual providing support within these contexts.

### Recruitment and Sampling

Participants were recruited through a community service drop-in space and existing staff networks. Interested young people met with the researcher to review the participant information sheet and self-select to an interview or focus group depending on their individual preference and comfort levels (Lynch et al., 2018). Sampling strategies were carefully considered as young people and their mental health involves both a sensitive topic and a population that can be excluded from research due to logistical or ethical concerns (Schelbe et al. 2015). This study used a combination of *purposive sampling* to ensure that young people were recruited with regard to age, gender, and ethnicity to ensure the inclusion of a diversity of perspectives and experiences (Bryman, 2012); and *snowball sampling*, which is a relational approach to recruiting participants, from within existing networks (Barbour & Barbour, 2003; Naderifar et al., 2017). Recruiting through established and trusting relationships was successful as it provided a proxy trust, which supported quicker rapport building and increased comfort, during data collection. In total, one focus group (n = 6) and fourteen interviews were completed with young people aged 16–19 (n = 5) years and 20–25 years (n = 9). Two participants withdrew before the focus group commenced and two focus group participants volunteered to do an interview, totalling 18 participants who completed this research. All participants provided written informed consent.

### Data Collection

When investigating complex health care phenomena, Vandermause (2007) has emphasised the need for innovative methods and combinations of qualitative methods that can support trustworthiness and quality in research (Creswell & Miller, 2000; Golafshani, 2003; Lambert and Loiselle, 2007). Data were collected from the young people within semi-structured interviews or a focus group. Interviews offered privacy and this study employed *intensive interviewing,* a CGT technique (Charmaz, 2014). Focus groups are a fun, interactive and developmentally effective method for gaining understanding about young people's views on sensitive subjects, such as mental health (Gibson, 2007). These combined methods contributed to data completeness, provided insight between the group (social) and the individual (subjective) realities, enhanced analysis as well as increasing participation (Lambert and Loiselle, 2007, 2008; Morse et al., 2002).

This research used *semi-structured* guides that were developed to explore the topic appropriately, sensitively, and flexibly (Galletta, 2013). Participants were asked to reflect on their service use experiences, before, during and after, and examples of these questions are presented in Table 2.

**Table 2:** Sample interview questions

Before	During	After
When did you decide to ask others for help?	Can you describe what the service was like to go to? What was the physical building/location like and how did that make you feel?	Were you satisfied with your experience/did it match your expectation?
Was there anything that caused you to wait longer/act quicker?	Can you describe to me your experience of working with a mental health practitioner/youth worker/pastoral care staff?	What do you think are the most important features that a youth mental health service should provide?
What was your experience like of asking this service/person for help?	Were you offered a choice in the type of intervention you would like?	Have you any suggestions that might improve the service?
Have you heard about other’s experiences of asking for help? If so, what were these like?	What did you need from that person in that moment?	Based on your experience would you go to a professional mental health service again?

Questions were also added on themes that were identified during data collection and posed to subsequent participants (Charmaz, 2014; Lambert & Loiselle, 2008). When the sampling technique was no longer yielding new properties to the phenomenon studied (saturation) a decision was made to stop data collection (Charmaz, 2014). This research used co-design and was conducted in partnership with young people (Richards, 2020).

Data collection took place in a community service location and interviews lasted between 20 min and 1 h 10 min. Participants were welcomed, offered refreshments and consent was reviewed. Participants were also offered time to settle in the room before commencing audio recording. Afterwards, participants were thanked and debriefed, and time was made available for participants to talk, ask questions and exit when they were ready. All participants reported having a positive research experience.

### Data Analysis

Charmaz’s (2014) CGT approach informed data analysis, a process that is embedded in all stages of this research. Data were transcribed by the principal researcher in sequential order and were uploaded to the Nvivo 12 software programme for coding and systematic organisation of data. CGT techniques such as memo writing, familiarisation with data and the constant comparative method were employed, which helped identify complementary, similar, or different patterns across data, within data and across data sets (interviews and focus groups) (Charmaz, 2014). The *initial coding* process was extensive and data were brought together into categories, sub-categories and concepts during *focused coding* (Charmaz, 2014) under the overall theoretical concept of “young people’s core needs in mental healthcare”. The data sets were analysed separately, but in the same manner, which resulted in complementary data analysis (Lambert & Loiselle, 2008).

### Ethical Considerations and Integrity in Research

Ethical approval was obtained from Ulster University Research Ethics Committee (UUREC No: 180010). Participation was voluntary without incentives, and informed written and verbal consent was obtained. This research was guided by The British Psychological Society's (BPS) *Ethical Principles for Conducting Research with Human Participants* (2014). Data were stored according to the General Data Protection Regulation (2018) and Data Protection Act (2018). A distress protocol was devised but not used and no participant withdrew data post participation. Participants under 18 years required parental consent to participate and were offered the option of having a chosen adult present during data collection. The principal researcher was trained in child safeguarding.

This research used dependability and trustworthiness checks including *member checking* for confirmability and *peer debriefing* (Creswell & Miller, 2000), a clear and transparent description of the design (Golafshani, 2003; Lambert & Loiselle, 2008) and rich descriptions of the data (Creswell & Miller, 2000), which can contribute towards transferability in similar regions with similar cultures and systems. In addition, r*eflective practice* using the guidelines form Creswell and Miller (2000), Galletta, (2013) and Charmaz, (2014) was used throughout the research process.

## Findings

This study analysed data from eighteen young people who took part in interviews (n = 14) and a focus group (n = 6) (Table 3).

**Table 3:** Young people’ demographics (n = 18)

Age	Gender	Location	Ethnic background	Education or employment status
16	M	Urban	Irish	Student
16	F	Rural	Irish	Student
18	M	Gaeltacht*	Irish	Student
18	Tm	Urban	European	Student
19	M	Urban	Polish	Student
19	F	Urban	Irish	Student
19	F	Urban	Irish English	Employed
21	F	Urban	Irish	Student
22	F	Urban	Irish	Employed
22	F	Urban	Irish	Unemployed
23	F	Urban	Irish	Unemployed
23	M	Urban	Black African	Employed
23	M	Urban	Black African	Employed
24	M	Rural	Irish	Employed
25	M	Rural	Irish	Employed
25	F	Urban	Irish	Employed
25	M	Gaeltacht*	Irish	Employed
25	M	Urban	Irish	Employed

*Gaeltacht, is the term used to refer to those areas of Ireland where the Irish language (Gaeilge) is the primary spoken language of the majority of the community

Demographic information was self-described by participants and this information has been deliberately disconnected from pseudonyms to further protect anonymity. All participants completed at least one full help-seeking episode with a practitioner (GP visit or counselling service), with most completing two to five to get their health care needs met, and those self-reporting the most severe forms of distress, including suicidality, tended to have the highest number (7+) of formal help-seeking attempts. Furthermore, most young people initially approached public services (GPs), turning to private services or community-based counselling when their needs were not met. It was noteworthy that young people (n = 4) from backgrounds involving migration or asylum seeking turned only to community youth services and pastoral care services in schools, avoiding formal services. Regarding the context of their problems, young people described seeking help from services for psychological distress related to low mood, depression, anger management, loneliness, anxiety, panic, trauma, self-injury, childhood caring responsibilities, parental bereavement, parents with mental health problems or addiction, family separation, refugee and migrant experiences, marginalisation, homophobia, transphobia, homelessness, childhood sexual abuse, suicidality and state care.

**Fig. 1:**
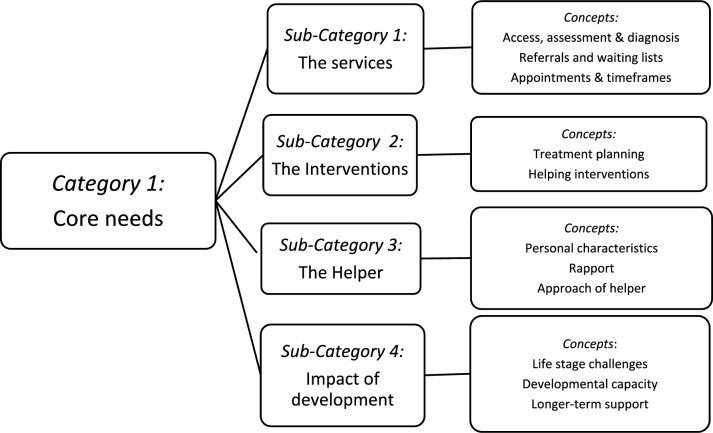
Young people’s core needs in mental health care

This section presents findings on the key elements that young people have reported as being central to their mental healthcare under the central category “core needs in mental healthcare”, which has four sub-categories: 1. *The services;* 2. *The helper;* 3. *The interventions,* and 4. *The impact of development* (Fig. 1).

### The Services

Young people described needing to feel welcomed by staff in a service, which helped reduce fears and anticipatory anxieties and increase comfort: “…someone to be friendly to you when you walk in a door can change your day” (Cathy). Furthermore, being provided with information about a service was important for supporting engagement: “Education on their roles and why they’re there and how they can help” (Thomas). Young people needed empathy and sensitivity during assessments: “Have a conversation, assess them without them knowing they’re being assessed” (Cathy), as well as opportunities to meet with a helper to ensure compatibility: “The young person has to learn about the person that they’re with and analyse what they are like, what do they like? how are they with them? are they comfortable in my presence? that’s very important” (Rachel).

Most young people described being highly distressed when they sought help with a service and highlighted the importance of a timely response and reassurance of help: “If they’re in crisis and you’re like “wait a week”, feeling like this? I might not be here in a week… there’s quite a lot of young people that, it’s bad, they just desperately need help right then and there” (Laura). Young people also needed access to appointment times beyond 9–5 pm, especially for those with low social support, such as individuals in state care, who often required out of hours support: “She would say any time you need me just call me and I’ll come down” (Rachel).

Regarding the environment, some young people needed helping spaces to be non-clinical, calming, and private: “She had a lovely wee art room with all these pictures and sculptures, it was so nice” (Josie). Others preferred multi-functional environments, such as youth services or public spaces, which helped them to manage stigma and discomfort: “It could’ve been in the youth centre, or we could’ve in a café, or we went to a park” (Áine). Young people with experiences of asylum seeking, migration or state care described trust as being connected to familiar community-based environments, where they could access longer term support: “…if it’s an environment that you trust, a positive environment, if you always go there, if they have helped you with problems before” (Andrew). Similarly, young people who identified as part of the LGBTI + community stated that they required explicit acceptance from services to support psychological safety, such as the placement of posters in receptions and waiting areas: “…if they’ve nothing up, then I’m not telling you anything!’” (Áine).

Finally, voluntary engagement and participation were described by young people as critical, with their autonomy appropriately supported at all stages of involvement in a service: “Choosing whether you want to even speak about something is more important than choosing what type of therapy you want…forcing somebody to talk when they don’t want to is actually more detrimental to their mental health” (Áine).

Fundamentally, how these reported needs regarding service factors were met or unmet greatly impacted how young people engaged with interventions and treatment.

### The Interventions

The main finding regarding interventions and treatment was that all young people needed and valued one to one support with a helper who, above all other approaches, techniques, and therapies, offered listening and offloading, especially during a crisis: “It was just having someone listen to me… that was all I wanted her to do, I just wanted to talk” (Gerard). Young people discussed wanting to be heard: “I just felt like I need someone to know” (James) and how regular offloading in of itself was often enough to manage distress: “I just needed the release of just being able to say all these things … and like the act of saying things out loud I think really helps” (Liam).

Prior to disclosures of distress, young people required time to feel safe which came from the opportunity for trust building: “People need time to open up to gain trust to feel like they can connect with the person they talk to” (Rachel). For many, a consistent, supportive and trusting rapport, was the intervention they needed most: “I needed, like this foundation level of ‘you’re not a horrible human being and everybody doesn’t hate you and you deserve to live’ and then I can get to writing things in books” (Erin). Several young people experienced the lack of a consistent rapport and seeing different professionals at appointments as distressing. “Like how can somebody get better if they see a different person every time?… where is the progression? where is the hope? where is the path forward?” (Joseph). For some participants who had little or no social supports, a single helper in a service could not meet all their mental health care needs and having opportunities to build a wider support network with access to multiple supports was important:

“I know if I rang [school counsellor] I know she would be there, and obviously [formal mental health support worker] is always there she still messages me, even if I went to the youth service, I know I would be supported no matter what” (Rachel).

Across youth, different interventions and approaches were needed at different times: “For some people talking therapy doesn’t work or it might work one week and not the other” (Áine). Opportunities and space for self-management were described as important, as were creative or artistic interventions or activities that could provide respite from distress, alongside support, especially in earlier adolescence: “…getting them into activities that take them away from what’s going on” (Cathy). Young people in later adolescence or emerging adulthood stated needing to learn additional skills and strategies for self-management and independence: “Sometimes you do need to learn how to live” (Laura). Some young people discussed needing support with understanding the role of pharmacological approaches and choice regarding whether they use medication as part of their treatment: “I was so young, I was so scared and I didn’t want to take this medication …” (Rachel). All participants described the prescribing of medication, specifically without an opportunity for talking therapy or life skills support as insufficient or inappropriate: “I don’t think any 14-year-old should be automatically put on an antidepressant” (Áine).

Finally, all of the young people stated that they needed clear boundaries around confidentiality established from the outset and for safeguarding concerns to be managed in an appropriate way: “… you need to make sure that the young person is 100 percent on board with confidentiality and that the person they are talking to is on board” (Claire).

Young people discussed how their needs for treatment planning and their ability to engage in interventions were significantly affected by the rapport and approach of their helper.

### The Helper

From the outset, young people needed helpers to actively create comfortable and safe environments: “Something as simple as, ‘I’ll make you a cup of tea’, straight away, you get a comfortable feeling” (Cathy). Young people also described needing helpers to inquire about well-being in order to work collaboratively and develop a plan based on individual needs for mental health care: “…do you think this will work for you? do you know what you need? and if you don’t that’s okay, there are other counsellors in the service that may do it this way or different ways” (Erin). Helpers who could communicate appropriately and relate well were reported as essential: “The person has to be very comfortable [with young people], they have to be able to have a good conversation … somebody who isn’t uptight, isn’t like scientifically smart about psychology” (Claire). The preferences for an established rapport versus a stranger before engaging in support varied, but all young people agreed that they needed a good quality rapport: “if there is no relationship between you and the other person, then the method of counselling is not going to work” (Joseph). All young people described needing open and authentic helpers: “…teenagers can tell when someone’s bullshitting them…” (Áine).

Many of the young people stated needing time to learn how to be in a professional supportive relationship: “… that was really important [time], some people find it hard to maybe express their feelings, they don’t know how to do it, what to do” (Robert). Central to rapport was trust, and young people with previous experiences of trust breaches from adults required a longer-term commitment of support: “It took me a very, very long time to open up to Mary. A very long time” (Rachel). Many young people reported that they also needed the rapport to feel ‘real’ and to know that they were genuinely cared for: “How do you expect someone to get better or to help them if you don’t know them? If you don’t actually care for somebody, how can you help them?” (Rachel). Genuine care was also experienced when helpers checked-in, provided after-care, or supported the young person in the search for the right support:

Lead researcher: “How did you know she cared?”
Áine: “Because she followed up”.

Genuine care could also be experienced through demonstrations of empathy and compassion: “…caring and compassion are basic human qualities, and it should be inbuilt, especially with people working in a mental health service” (Joseph). Many young people also reported needing positive attention: “Because you do need a bit of attention at the time!” (Laura). In addition, non-judgemental and understanding helpers were discussed as central to mental healthcare: “Give me a way where I can get it out without worrying about your reaction” (Laura), especially for young people identifying in the LGBTI +  community: “I think I needed someone to really genuinely understand and just accept me the way I am” (James). All young people reported that they needed to feel respected, validated, and that their distress was taken seriously: “At the start I needed her to tell me I am not crazy” (Claire) and they did not need to be stereotyped, othered, patronised or dismissed: “I didn’t want it normalised what I was going through, I didn’t want someone to say ‘aww it’s just a phase and you’ll grow out of it, you’ll be fine’” (Cathy). Some young people also reported needing a helper who could understand how mental health affected the ability to attend appointments: “…sometimes you’re too sick to see people and you’re like I can’t deal with talking to you… I’m too depressed and too tired and there’s no point, I just don’t want to do anything today” (Áine). Young people described the positive impact of helpers who could encourage personal power and provide hope and reassurance: “It's them counsellors that give you hope and make you feel like there is a way forward… you need what every kid needs that’s struggling—you need hope” (Joseph). This was especially important when a young person was experiencing family conflict or distress from romantic relationships: “To speak to someone that was entirely impartial and there for me and not anyone else was what I needed at the time” (Gerard).

One participant, Richard, described needing more than a single helper for connection, acceptance, and community:

“real people that sit down and chat to you and take time out of their day to ensure you’re okay, everyone wants to feel like they matter… if you walk in here and somebody remembers your name… and have a conversation with you, it’s huge, in fact it’s helped me”.

Young people from African backgrounds stated that they had different needs due to their life and migration or refugee experiences: “I don’t like talking about like, as if Africans are different, but it is different” (Thomas). Thomas also stated that he would not use formal services, needing the type of community networks and established relationships typically found in schools and youth services: “… for a young African, it would be environments where they can build relationships… I feel like through youth services is probably the best way … because without, for me, I would have never [sought help]”. Thomas stated that young people from African backgrounds who have experienced migration or asylum seeking are likely to have other stressful or traumatic life experiences, including racism and discrimination. He needed an appropriately experienced or trained helper who could validate distress: “…it’s much harder, it’s because people have witnessed so many things from a young age, it builds up and it’s like [pauses] nobody ever [pauses] and our parents… talk to us about it as if it’s a myth. (Thomas)”.

Overall, this study found that helpful rapports supported young people to stay in mental healthcare and were based on a helpers’ understanding of a young person’s developmental needs.

### The Impact of Development

There was a consensus from young people that being young could be a challenging and lonely time. No young person described growing *out* of their distress from childhood but growing and changing *with* their distress: “All the stuff you have been experiencing since you were a child comes to another level of understanding” (Erin). Many of the young people described needing guidance, as labelling and articulating their healthcare needs was difficult because of their age: “I kind of knew it but didn’t, I just needed someone to help me to open up and realise what was going on with me…” (Claire).

Young people reflected on how their mental health needs varied and how these changed across their youth:

“you’re not the same person that you are at 16 and 20, or even 10 or even at seven, whenever it first started… so you have different needs and somebody at 20 could have the same needs as somebody at 16” (Cathy).

The age of 18 years was described by most as a “crucial life stage”, representing an important developmental nexus due to their important legal transition. Young people reported being expected by caregivers and other adults to achieve academically and become independent during this stressful and pressurised stage, which was described as compounded by mental health problems:

“It is extremely hard for young people because everyone has this idea that young people have to go into school, get their leaving cert [school qualification], go to like university, get a good job, have a nice house, have a nice family… this is the way you have to do it and if you don’t, then you are a lost cause” (Rachel).

For young people who transitioned to university, the experience was intense, and they needed mental health care to incorporate mental health education, relationship guidance and general life support: “At 16 it’ll be okay, but when you’re first trying to find your feet in the world … it can be rather overwhelming” (Liam). The move to emerging adulthood around twenty years of age was described as a time for improving self-management strategies in line with a drive for increased autonomy: “I needed someone to help, to support me in getting better myself” (Erin). Liam discussed needing life experience to be able to engage in CBT, which can be harder to obtain in earlier adolescence: “like it gives you the tools to deal with it and they try to teach you how to do that, but I do think that you do need some amount of life experience”.

Young people who had accessed help on either side of the age divide within public mental health services, described children’s services as patronising for adolescents and discussed how the move to an adult category was not always developmentally appropriate, and the need for services to address this:

“… cutting off the care at 18, moving you into a different bracket? That’s not a good idea because you are not a fully formed person yet and your brain is still developing, like – **huge**, different changes, in like your emotional well-being” (Áine).

Some young people described using multiple services across their youth to meet their need for longer term support: “mammy said ‘maybe you need something long-term’ and I thought oh my God, and everything just kind of clicked” (Claire). Longer-term support was crucial for young people who experienced state care because professional relationships became a proxy for family style relationships: “She was the only person, like when I talk about her it touches my soul … no one in my life has helped me that much” (Rachel).

Young people living in a family environment with conflict or abuse needed their adult helper to provide support, safety, and perspective: “I wanted someone to say what your dad is doing is wrong, and it is okay to feel like it’s wrong, and there’s nothing wrong with you” (Erin). When distress was connected to a caregiver relationship, the gender of the helper became a need in support: “Whereas daddy and that man were both men and automatically I’m kind of like closing up because I don’t want to open up to another man” (Claire). Some young people described being protective and defensive with helpers in earlier adolescence as a result of poor-quality relationships and experiences of adult rejection:

“When you are that vulnerable and you feel like you hate yourself, you’re looking around for other people to hate you or for other people to think negatively towards you… you are looking for signs of you being a burden” (Erin).

Young people’s development impacted the way in which they engaged with healthcare and many required flexible time, length and frequency of appointments. Some reported needing consistent and predictable appointments, while others described needing support, as and when problems arose, or intense support during crises: “…there was a point where I would have to go like maybe twice a week” (Claire). Younger adolescents needed endings managed slowly and carefully: “…and I remember I cried when it was our last session because I almost thought of her as a friend because I was telling her all of this and then looking back, I think, I was so naïve” (Claire).

In summary, this section presented data across four sub-categories: 1. *The services;* 2. *The helper;* 3. *The interventions,* and 4. *The impact of development.* These findings on young people’s experiences with mental health provision highlight the distinct needs young people have, which will now be discussed.

## Discussion

This research found that current mental health care provision varied across formal and semi-formal service providers due to the individual approach of practitioners, policies and the funding structures. Participants’ experiences across all services were examined, evaluated and analysed together to map the key features that were discussed as *helpful* and *unhelpful*, which were found in all services to different degrees (Table 4).

**Table 4:** Youth core needs in mental health services

Service	Approach & Rapport	Interventions	Developmental
Welcoming staff and reassurance of help	Safe, approachable, and friendly	Individualised face to face support	Separate service for young people
Information and expectation setting	Opportunities for trust building	Age-appropriate interventions	Longer-term access and support
Direct access and timely response	Confidentiality and privacy protected	Hope, reassurance and positive attention	Opportunities for rapport development prior to intervention
Option of helper	Collaboration and planning	Foundational skills for participation	Opportunity to develop conceptualisation of mental health
Voluntary participation	Non-judgemental and empathetic support	Culturally appropriate interventions	Scaffold learning through modelling formal help-seeking skills
Appropriate assessments	Helpers who listen and validate experiences	Flexible, adaptable, and creative methodologies	Advocacy and guidance with age-related or general life challenges
Flexible service times	Relatable and suitable helpers	Consistent care	Access to a support network (clinical and non-clinical)
Policy that supports interventions	Provision of genuine care	Self-management spaces	Non-stigmatised spaces
Policy that supports families	Appropriate professional boundaries	Activities that promote respite	Support with psychopharmacological interventions
Inter-agency and multi-disciplinary service	Appropriately trained and experienced helpers	Opportunities for building community	Age-appropriate expectations for service use including ad-hoc, routine and crisis support

Young people have varied life and healthcare experiences (Höylä, 2012) and the intention of this research and the data presented in Table 4 was not to provide a reductionist list of facilitators to healthcare but to communicate the core needs for mental healthcare as reported by young people in this study with regard to *service factors*, the *approach* and *rapport of the helper*, the type of *intervention* and respect for *developmental* capacity.

### The Role of Services in Developmentally Appropriate Care

All service provision is shaped by the wider policies governing it and there is an urgent need to evolve youth mental healthcare beyond *youth friendly* labels. Services can become truly developmentally appropriate, and *youth centred* (Rickwood et al., 2019; Sawyer et al., 2018) with suitable environments, integrated services and specialist youth trained staff (Dopp & Lantz, 2020; McGorry et al., 2019), an approach that aligns with community mental healthcare approaches of person-centredness, earlier interventions and life course perspectives (Sowers et al., 2022). Youth centred services can consider supporting the essential role of caregivers, who are managing other responsibilities, such as employment and childcare, and who provide essential resources for young people to attend healthcare (Lynch et al., 2023; Thornicraft et al., 2016). Services can also consider how their policies can result in the exclusion of groups of people who traditionally experience marginalization from statutory services (De Anstiss & Ziain, 2009; Fanning, 2012; Masuda et al., 2009) and ensure that service design includes a diversity of perspectives (Sowers et al., 2022).

This research found that ending service provision at 18 years of age is misaligned with life stage needs (Solmi et al., 2022; Arnett, 2023), a practice described by McGorry et al. (2019) as an “anachronistic and developmentally inappropriate ‘hard border’” (p. 140). Eighteen years of age was found to be possibly the most harmful time during youth to end or transition mental healthcare provision, especially for young people in state care. This transition was re-traumatising due to the loss of supportive professional relationships, who were essential parts of a young person’s network, and upon whom they depended on for ongoing connection, comfort, and care beyond 18 years of age (Stein, 2006). Services can improve their alignment by providing *youth mental health services* to all in the life-stage of youth, approximately 10–25 years, reserving child services for children and adult services for those over 25+ years (McGorry et al., 2019; Westberg et al., 2020).

Access to public mental healthcare in Ireland is unnecessarily complex and does not support self-referral, which is practiced in most community or private services (HSE, 2021). Young people with significant distress described waiting periods in formal systems as between 2 months to 4 years after asking for help. Waiting lists were found to be particularly harmful and were generally connected to service underfunding and inefficiency (Barnardos, 2017; Dopp & Lantz, 2020; Fargas-Malet & McSherry, 2017). When young people do obtain access, it is critical that they are welcomed and provided with information regarding expectations, options, timescales, staff roles and limitations, how their information is stored, and how confidentiality is managed with age-appropriate examples (Hackett et al., 2018; McGorry et al., 2019). An important pillar of community mental healthcare is the promotion of the right for individuals to be included in treatment decision making (Sowers et al., 2022; Thornicraft et al., 2016) and this research found some concerning descriptions of professional practice that did not prioritise consent nor include young people in decisions about their mental health care. Without voluntary participation, interventions and outcomes can be at best partially successful and at worst harmful (Damien et al., 2018). Furthermore, respecting and leveraging a young person’s developing autonomy was found to be therapeutic and a priority for young people (Wilson & Deane, 2012) supporting recovery-orientated and person-centred perspectives (Sowers et al., 2022; Thornicraft et al., 2016). Being in the life stage of youth impacts how mental health is understood, labelled and how help is sought. The current logistics involved in accessing healthcare are part of a *formal help-seeking skillset*, that is outside developmental capacity and rarely mastered before adulthood. Accordingly, developmentally appropriate mental healthcare can also include accommodating and supporting ad-hoc or crisis support, which can be an expected and normative help-seeking style in youth (McGorry et al., 2019; Rickwood et al., 2019; Thornicraft et al., 2016). Mental health systems which use a 9–5 pm service-centred paradigm can have high cancellation and no-show rates, demonstrating further inefficiency and unsuitability. Thus the prioritising of service users’ needs rather than service providers’ needs could contribute to reductions in these burdens, wider system issues and increase efficiency (Sims et al., 2012).

Young people require informal approaches at all levels of service provision (Davison et al., 2017). Helpers can use first names only and exclude the use of academic or medical titles, which serve no purpose for young people other than to reinforce power structures and undermine partnership. Assessments, depending on the helper’s empathy, were found to have the potential to be either violating or part of a trusting foundation on which to support engagement and provide intervention. Furthermore, clipboards, administration, and notetaking were described by young people as intimidating, distracting, and unnecessary. The retelling of life stories and psychological distress to multiple staff was found to be a common yet harmful practice and young people need the individual that completes their assessment to be the helper that they will continue with (Hackett et al., 2018). A small but important finding regarded the gender of the helper and how this can impact engagement; if a young person’s distress came from conflict with a caregiver of the same gender, then engagement could be affected by this factor (Pearson & Hyde, 2021).

The clinical spaces and waiting rooms at formal services were found to be frightening, intimidating and unfriendly to young people (McGorry et al., 2019), often intensifying anticipatory anxieties, and contributing to feelings of defectiveness, stigma, or alienation. Young people feel safe in comfortable settings and value refreshments, such as warm drinks or snacks as these can communicate nurture and care, and mimic features of informal help-seeking, which was reported as increasing comfort and lowering anxiety. Young people, especially in early to late adolescence, prefer multi-functional community-based environments, as these spaces manage stigma, fears, and discomfort better than clinical settings, and were found to be the least harmful service environments. Having access to a positive and safe physical environment in itself can be therapeutic, especially to young people experiencing marginalisation, state care, migration, asylum-seeking or homelessness (Crosby et al., 2018). Young people with low social support need community services, specifically youth services, for supporting and maintaining them in mental healthcare as youth workers can assist with problem-solving, as well as advocating for health care needs, and supporting personal and social development through encouraging interests, community building and facilitating peer connectedness (Rickwood & Mazzer, 2012; Rickwood et al., 2005; Harland et al., 2005). This promotion of a wide network of supports and services who can adequately provide care to a young person is both supported by community mental healthcare approaches and is what young people in this research reported as wanting and needing (Sowers et al., 2022; Thornicraft et al., 2016). Young people who identify in the LGBTI +  community need explicit acceptance, such as visible placement of posters or social media posting, which can reduce some of the anxiety and fears regarding stigma and discrimination (Fish, 2020). This research also found that young people in emerging adulthood were able to manage formal spaces better than adolescents, especially when helpers were appropriately trained but in general, all participants preferred community spaces. The concept of therapeutic spaces for young people can also be revised to include firstly, unstructured spaces for self-management in schools and youth centres and secondly, formal support outside of a room or a building, such as a park or a public space, for young people who would prefer that (Tillman et al., 2018). Services can consider how and when their physical design reinforces stigma and secrecy, or supports privacy, openness, and acceptance about mental health (McGorry et al., 2019).

### How Young People Want to be Helped

It was found that young people need one-to-one individual support unless they request otherwise. Therapeutic group intervention as a default or substitute for individual sessions, often as a result of underfunded public systems, are not appropriate unless requested. Moreover, young people often first need to be supported to develop the foundational skills required to communicate and participate in such activities (Lynch et al., 2021). This research found that listening is the single most important intervention young people need, with opportunities for emotional offloading being associated with increased self-management between meetings. CBT approaches were found to be somewhat helpful in emerging adulthood but in earlier adolescence these could lead to young people doing exercises to gain approval from their helper. Pharmaceutical interventions were the least valued, but the most common, and findings suggest that young people need support in understanding their specific role in treatment as well as how to manage taking medicine and undesirable side-effects, which were described as interfering with other self-management and coping strategies (Draucker, 2005). Additionally, young people need to be listened to when they do not want to take medication and want a relational intervention instead, which is essential in avoiding marginalising young people further and in supporting person-centred approaches (Sowers et al., 2022).

Central to young people’s care is access to accurate life-stage information and guidance around relationships, sexual health, education, employment and accommodation, and any other developmental related concerns, such as social, economic, or legal support (Harland et al., 2005; Rickwood et al., 2019). Youth is a time of constant change (Best & Ban, 2021) and interventions such as listening, guidance, and skills building are needed at different times, across the life stage (McGorry et al., 2019). Short interventions, adapted from adult models, do not meet the needs of young people and appropriate interventions need to be responsive to the reality of how problems arise and are managed in youth (Hackett et al., 2018); firstly, through access to longer-term and consistent supportive relationships up to the age of 25 years approximately and secondly, during crises or difficult transitions which can require more frequent and longer support meetings. Furthermore, as mental health distress can affect engagement and attendance, services can empathetically support young people with increased flexibility regarding attendance, and the removal of punishing policies that discharge young people for repeated no-shows without consultation. When mental health is relatively stable, support can move between routine appointments or check-ins and open offers of support. Young people should not be discharged because they are doing well but offered information on how to return for support with future challenges. Sometimes the nature of problems in youth can require more than one supportive adult and young people can benefit from interdisciplinary approaches using a wider network of clinical and non-clinical supports (McGorry et al., 2019; Thornicraft et al., 2016). In the absence of integrated mental healthcare, young people use multiple siloed services in an attempt to get their needs for longer-term support met (Damian et al., 2018; Dopp & Lantz., 2020) and this was a common finding in this research for young people with much adversity, such as those with experiences of homelessness or state care. Services should anticipate this reality and ensure trauma informed care includes the implementation of multiple supports across settings so that young people with severe distress and low social support are not re-traumatised through the loss of supportive and consistent relationships (Thornicraft et al., 2016; Damian et al., 2018).

### The Helping Relationship

Supportive rapports and person-centred approaches are central to young people’s mental health care (Lynch et al., 2021). In keeping with their developmental capacities, young people want to be liked and to receive positive attention from a helper, especially when they appear to be resisting relational connection, which expresses a need to feel safe. Active listening, inquiry, demonstrations of warmth, reassurance, and hope from helpers alongside collaborative and individualised approaches far surpassed any specific intervention or medication (Hackett et al., 2018). These positive relational exchanges supported comfort, safety, and trust building, encouraging feelings of connectedness, and being helped (Davison et al., 2017; Persson et al., 2017; Wilson & Deane, 2012). Moreover a consistent, safe and supportive relationship was found to support young people to develop positive self-image, reframe illness and develop self-management skills further (Thornicraft et al., 2016). Findings show that switching up service staff, which was reported as common in public systems, undermined trust in services. Additionally, this practice can be experienced as objectifying, with support based on case management and not on rapport, partnership or trust, a topic of central importance in this research. Many participants described having previously had their trust broken by adults in positions of trust, including family, teachers, social workers and other mental health professionals and thus needed time for it to establish with a helper in a service (Draucker, 2005; Jones et al., 2017). Young people from migrant or refugee backgrounds often only sought help in schools or community services with helpers they had developed trust with. For all young people, this process of trust building was supported by time and space for the young person to “assess” their helper, and for rapports to feel “real”, and was often only achieved when professionals were described as balancing looser professional boundaries with professional responsibilities, and in some instances breaching service policy to ensure basic care needs were met (Ungar et al., 2018; Jones et al., 2017). This finding indicates the need for professionals to be supported by appropriate youth-centred service policies that assist them to do their work without having go outside service policy to meet a young person’s mental health care needs.

Time was also connected to young people’s ability to articulate or understand their distress and health care needs were often only revealed as trust formed and helpers assisted with psychoeducation and communication skills (Simmons et al., 2011; Ungar et al., 2018). Helpers also need to be able to relate to the internal challenges of mental health and provide compassion and empathy on how attendance can be affected by distress. Young people can also need support with service use and helpers can scaffold learning by supporting the development of the skills needed to negotiate services, treatments, options, and processes, in addition to being transparent and forthcoming about expectations and limitations (Jones et al., 2017). It was found that young people implicitly knew what they *did not* need, or what was not working for them, and wanted to be supported to ask questions and try out different interventions (Jones et al., 2017). Expectedly, young people are sensitive to familiar adult–child power imbalances and helpers can pay attention to undue influence or control over the direction of support by communicating appropriately, and being attentive to their manner, body language and dress. All young people can naturally become attached to those who offer comfort and support, and so endings or transitions need to be managed sensitively, especially in early to mid-adolescence.

Confidentiality was another central topic (Lynch et al., 2021) with young people reporting the need for clear boundaries, protected confidentiality, privacy, and that child safeguarding is managed in a calm, responsive and somewhat predictable manner. Young people can need repeated explanations of confidentiality to reach full understanding of the context of their support and the consequences of disclosures. Helpers also need to prioritise the provision of confidential and empathetic spaces for young people who experience compounded stress, for example those who have low social supports, have experienced trust breaches or multiple traumas in childhood, marginalisation, discrimination or racism (Cniro et al., 2005; Collins & Barker, 2009; de Anstiss & Ziaian, 2010). When young people can experience a consistent supportive rapport, and they feel respected, listened to, validated, and cared for, they can often begin to address their mental health problems (Lynch et al., 2021).

## Limitations

This study has some limitations. Firstly, while this research included young people from diverse backgrounds and from groups that typically experience marginalisation, young people from other important communities, such as the Travelling and Roma, were not recruited (Fanning, 2012). Secondly, it is possible that unconscious researcher bias could exist despite the provision of rich data excerpts, use of reflective practice, and the inclusion of a transparent description of the methods. Finally, the findings are intended to be culturally specific and while this research took place in a high-income country which shares similar mental healthcare systems as other Westernised countries, there could be limitations due to its size and geography.

### Implications and Recommendations for Further Research, Practice, and Policy

This research has two primary recommendations, firstly, findings suggest that formal mental health services (public, private, community-based) are not adequately meeting young people’s needs and thus need reform or revision to reduce health disparities. Services can unintentionally have harmful policies, practices, and approaches, and these were particularly pertinent to public services, where the highest quantity of negative and harmful experiences were reported. This research has identified the *core needs* that young people have reported to be essential in mental healthcare and has provided guidance (Table 4) on factors that service providers and practitioners can attend to when considering how a service is designed and delivered. Providers can consider how to reduce harmful practices when providing therapeutic interventions to young people, specifically with regard to developmentally appropriate provision, and ensure that service policy prioritises the rapport and approach of the helper along with the choice of intervention and involvement of the young person in treatment decision-making in a safe and suitable environment. However, the current siloed approach of the public sector to youth mental healthcare in Ireland, and across many regions, has created self-limiting infrastructure which impacts upon what can be realistically achieved by individuals and practitioners alone (Dopp & Lantz, 2020). Complete reform of public youth mental healthcare is necessary, and it is advised that the findings from this research are considered in planning and co-design whilst considering local, regional, and cultural practices as well as findings from international research (McGorry et al., 2019; Rickwood et al., 2019).

Secondly, researchers can use co-design to support young people to take part in research and communicate personal experiences of help-seeking and service use. In the interest of meaningful early intervention, engaging policy makers and service users in co-design research is critical (Forde et al., 2018) for ensuring services are appropriate and relevant, both culturally and regionally. Additionally, further research could continue to include young people with lived experiences of mental health and help-seeking to build on the findings from hypothetical studies. Furthermore, through alternative approaches, such as participatory action research (McIntyre, 2007), future research could strive to include young people who live in communities that experience marginalisation and are typically excluded from research.

## Conclusion

This research explored young people's (aged 16–25 years) mental health experiences (n = 18) using a Constructivist Grounded Theory approach (Charmaz, 2014) and rich data were analysed regarding the shared lived experience of being a young person and asking for help with a mental health problem. Qualitative evaluation of experiences supported the identification of the core needs young people have reported to be essential for their mental healthcare and in the interest of meaningful early intervention, reform or revision to ensure services are developmentally appropriate is now necessary across youth mental health services in Ireland, specifically in the public system. To reduce health disparities affecting young people, services can also ensure that rapport and approach of the helper are prioritised along with the choice of intervention in a safe and suitable environment. The findings of this research demonstrate the importance of youth participation in the co-design of service provision, which can ensure that services provide relevant, respectful and suitable care that reflects the way in which young people experience mental health problems as well as the ways in which they want to be helped.
